# Supplementary material for: Bridging the gap: Using reservoir ecology and human serosurveys to estimate Lassa virus spillover in West Africa
Source: PLoS Comput Biol. 2021 Mar 3;17(3):e1008811. doi: 10.1371/journal.pcbi.1008811 (PMC7959400; doi:10.1371/journal.pcbi.1008811)
Supplement: Attachment — Submitted filename: PCB_Lassa.pdf [file pcbi.1008811.s002.pdf]

Bridging the gap: Using reservoir ecology and human serosurveys to estimate Lassa virus incidence in West Africa

Reviewer summary

This paper presents a method to predict zoonotic spillover risk from environmental data. Boosted classification trees were used to model the relationship between environmental variables, the reservoir population and animal Lassa virus prevalence to ultimately predict the incidence of human Lassa virus infection. The authors propose that the methodological novelty lies in the distinction between the vector distribution and pathogen reservoir layers (used to generate predictions) and the human seroprevalence layer (used to model the link between predictions and observations). The model predicts a far greater number of Lassa virus infections than current, outdated estimates and predicts their distribution across countries in sub-Saharan Africa. These results imply that existing surveillance likely misses the majority of symptomatic and asymptomatic infections and highlights key geographical areas for enhanced virus surveillance. Much of the discussion and implications pertain to future work and routes to understand the (often substantial) remaining variance between predicted and observed human seroprevalence. To me, the main hypothesis being tested is "does the presence of Lassa infected *M. natalensis* predict magnitude of human seroprevalence?", and the results suggest "not very well on its own".

I am not an expert on zoonosis nor Lassa fever, so I am reviewing this from the perspective of an interested infectious disease epidemiologist with a computational background. I apologize if my questions or comments on the disease natural history seem obvious or silly!

The paper is very well written; the flow between sections is great, the introduction sets the scene nicely, and I left the methods feeling qualified to understand the remaining sections. The code sharing and accompanying readme are clear, and I was able to re-run their analyses with no problems (though I do not have a Mathematica license).

This paper appears to be an important next step in a fairly sparse literature (at least from my cursory searching). However, right now it is predominantly an eloquent presentation of a method (with some novel ideas) but with only a cursory consideration for the biological implications. I am also a little confused by the methods: combining the two layers by multiplying them, then using the binary classifier score as a predictor for a continuous outcome in the GLM seems like a weird pipeline. Though this may be my misunderstanding. Given that the binary classification score (from the first two layers) appears to have little explanatory power in the GLM, I am not sure what the results add over the referenced Mylne et al. paper. I think these methodological concerns need to be clarified and the implications of the results (rather than just the unrealized potential of the method) made clearer if this is to warrant publication in PLoS Comp Biol.

Major comments

- The authors claim to be modelling spillover risk, but I am not convinced that is what their model is predicting. I am probably missing something crucial, but it

seems odd to train the model on binary data (presence/absence of LASV or *M. natalensis*) and to then use the predictions as a quantitative estimate of spillover risk. The model may predict a high classification score for presence of LASV infected rodents in a pixel, but does that necessarily mean that it is predicting higher LASV prevalence in the reservoir population? Is it distinguishing between a small rodent population with low prevalence and a large population with high prevalence? It seems like those data points are weighted the same here. Therefore, from what I understand, the method is only predicting the binary presence or absence of at least 1 LASV infected rodent which is what Mylne et al. did (they call it suitability index rather than spillover risk). If so, what do the authors think the GLM would look like if Mylne et al's suitability index were used instead?

- The GLM clearly does work given that it explains some variance and the slope is significant. However, if the predictor is only binary, then isn't the comparison to magnitude of human seroprevalence somewhat flawed? For example, a classifier for "absence, low LASV prevalence in rodents, high prevalence LASV prevalence in rodents" would make more sense if the aim is to predict magnitude of risk. Is a lot of quantitative information being thrown away converting to a binary prediction, and is this why the GLM appears to explain such little variance? Maybe I am trying to be too mechanistic, but I think this would be helped if the methods and results clarified why this approach makes sense.
- Figure 2 and associated methods: I have been trying to intuit why simply multiplying the likelihoods makes sense, but something feels off. The D_M layer is predicting the likelihood of *M. natalensis* presence given the environmental variables, whereas the D_L layer is predicted the likelihood of LASV circulation given the environmental variables **and** the presence of *M. natalensis.* I would recommend adding some text to explain the intuition behind this. A bit of a half-baked thought, is it related to the conditional probability P(LASV | rodents) * P(rodents)? If so, is multiplying the likelihoods the right approach?
- Discussion of the implications: does this new denominator change our understanding of the Lassa fever clinical spectrum and epidemiology? For example, if the IFR is 2%, then 3 million infections implies 60,000 deaths. Is this consistent with underreporting of LF-associated deaths, or does it imply that the IFR is set too high?
- A positive comment – combining a SIRS model with predicted seroprevalence and immune waning estimates to get incidence is really neat!
- Figure 4 is pretty underwhelming: it leaves the reader feeling unconvinced that the modelled relationship is real or useful. The plot should at least have confidence intervals on the model line. Also is this from the weighted or unweighted version? And relating to Figure 5 (see minor comments), is this actually doing much better than assigning each pixel the mean seroprevalence? The authors derive a null model for Lassa seroprevalence but don't really use it in this way.
- The discussion is well written and raises a lot of interesting points. However, it does not discuss the results from the D_M and D_L layers at all. For example, it is not clear how to interpret the result that maximum precipitation and

precipitation contingency are the 2 most important predictors in both layers. Are the key environmental predictors the same or different to previous work, and are there any novel biological insights there? L467 hints at this, but I didn't feel like the discussion clarified what these insights are. There is some text in the supplement, so this should at least be referred to.

Minor comments

- The omission of a time component in the model seems important. The reader doesn't know when e.g., the rodents were captured relative to e.g., when seroprevalence was measured. Is there some evidence to support the assumption of steady-state seroprevalence rather than increasing seroprevalence over time? Are there any longitudinal or repeated cross-sectional studies?
- I think there is an opportunity to further test the sensitivity of the D_M layer. You have an independent dataset for true positive *M. natalensis* presence: the data used to train the D_L layer. Does the D_M layer accurately predict the presence of *M. natalensis* in those locations? This might be a useful small supplementary analysis to support the accuracy of the D_M layer.
- The AUC for the same predictors (absence/presence of *M. natalensis* or Lassa) looks almost identical to those in Table 2 of Mylne et al. Does this mean anything, given that the top environmental predictors are the different?
- L41: It isn't clear to me how the issue of human seroprevalence not coinciding spatially with location of infection is resolved here. When comparing the model predictions to observed seroprevalence in the GLM, is this issue not being re-introduced anyway? Consider clarifying.
- L70+ The date range for the animal and LASV data is not mentioned, but it is for the human seroprevalence data. Would be good to see this to understand how comparable the dates are.
- L70+: Similarly, the survey data are not described in much detail. I understand that there are so many sources that this would take up too much text, but a supplementary table, particularly for the human seroprevalence data, describing sample sizes, demography, protocol etc might be useful.
- L121: The text suggests that the human seroprevalence data are surveys of all arenaviruses antibodies, not specifically Lassa. Perhaps this is obvious to someone with experience with these pathogens, but is there a reason why the included studies were not LASV specific? Are the assays highly cross-reactive or are there no other arenaviruses that infect humans? This should be clarified.
- L130: I understand the decision to only include land types that have been consistent for 20 years from a methodological perspective, but does this not ignore a major source of spillover risk – increased activity at the animal-human interface? It's not my area so I may be wrong, but if this is important, its implications and limitations should be discussed in the main text. From the abstract of the cited Gibb et al. paper: "Although the recent increase in LF case reports is likely due to improved surveillance, **recent studies suggest that future socio-ecological changes in West Africa may drive increases in LF burden**".

- Related, L444 mentions time but not in the context of land type. Perhaps mention this here.
- L177: Is a Bonferroni correction for multiple comparisons appropriate here? Probably not given that this is just an arbitrary pre-filtering step, but something to consider.
- L207: Maybe I'm being slow, but I don't follow the logic for how false negatives make the classifier conservative. Please clarify.
- L209: Dumb question, but is this spatial distance or minimum distance across environmental variables?
- L258: Is it not a fairly easy addition to the model to assume some deaths? 2% mortality is a lot of deaths when you're predicting millions of infections! How would this relate to how we understand LF epidemiology now? Though I understand if this would mess up eq 2-4.
- L266: is there evidence that arenavirus seroprevalence is at steady state or is this an assumption?
- L280: Refers to the data section, but there is no information on the WorldPop data there. Is this text missing or is the reference erroneous? (I think it should refer to S1 Appendix).
- L282: Assumed a mean lifespan of 50 years for all locations. Why not use country specific estimates?
- L281: It took me an embarrassingly long time to intuit these equations. The equations are correct, but I couldn't get my head around the idea of there being a constant "steady-state population size" if the real population size is growing (ie. when b > dN). I think I see now that this is a possible equilibrium because i) the incidence rate does not depend on the I compartment (as in a textbook example) and ii) crucially, the birth rate b is assumed to be **not** density dependent. I couldn't understand how the incidence of new infections could be constant while the population is growing and the proportion in each compartment stays the same. But I think it works because b just feeds in a constant number of new susceptibles (rather than an increasing number if the term were bN, which I think would be more standard). Nothing to be done if this is a trivial realization, but some text to explain the intuition for why this steady state exists and the rational for a flat birth rate might help a confused and pedantic reader like me!
    - I found page 16 of this: https://arxiv.org/pdf/2004.04675.pdf and this paper which might be helpful: https://doi.org/10.3390/math5010007. Not suggesting to cite.
    - Also page 88 of the "an introduction to infectious disease modelling" by Vynnycky and White was useful.
- L292: At the moment, the authors present the immune waning estimate from McCormick et al. 1987 as an upper bound. Is there any evidence to suggest that waning might be even faster than the point estimate from McCormick 1987 (e.g., were there confidence intervals on lambda in that paper)? If so, this would be a useful scenario to present.
- L319: missing space before the bracket.
- Figure 5: a histogram of residuals might be a useful subplot here. It's hard to assess how well the model is doing from this plot.

- L376: This is a really key paragraph, but a bit more information on the surveys, for example, predominant housing type in particular outliers above/below the prediction line, could be useful. Was this information not available to include in the GLM here?
- I don't know this literature, but it looks like there may be a few relevant studies tackling a similar problem that are not discussed, e.g., https://besjournals.onlinelibrary.wiley.com/doi/full/10.1111/2041-210X.12549 which also provides quantitative spillover estimates

Comments on code
Really nicely documented, well written and clean code. Great job. I ran everything line by line and found no obvious bugs! Only small comment is to not use reserved words (eg. data, grid) for user defined objects.

Generate_Reservoir_Layer.r
L79: in the object `classi.dat.rod`, none of the pseudo-absence variables have countries or confidence. Just checking that this doesn't impact anything downstream.
