# Supplementary material for: Bridging the gap: Using reservoir ecology and human serosurveys to estimate Lassa virus spillover in West Africa
Source: PLoS Comput Biol. 2021 Mar 3;17(3):e1008811. doi: 10.1371/journal.pcbi.1008811 (PMC7959400; doi:10.1371/journal.pcbi.1008811)
Supplement: Attachment — Submitted filename: Response_To_Reviewers.pdf [file pcbi.1008811.s003.pdf]

Dear Dr. Amy Wesolowski and Dr. Nina Fefferman,

We're grateful for your roles in the review process, as well as the helpful comments provided by the reviewers. As suggested on the PLOS website, we've provided the revised manuscript, as well as a version that indicates added text (blue) and deleted text (red). For this round of revisions, we've removed figures from the main text and uploaded them as separate files, and we've adjusted figure and table legends to be in accordance with manuscript guidelines. We've tried our best in the point-by-point response to clearly describe where changes have been made in the manuscript. We hope these changes have made the manuscript acceptable to PLOS Computational Biology. Please let us know if any other revisions need to be made. All revisions to the datasets and code have been pushed to GitHub in the "rev1" branch of the pathogen-spillover-forecast repository [1]. We start by addressing a key issue raised by multiple reviewers. Next, we reply to major revisions or comments that were shared by multiple reviewers, as well as revisions that changed the model or the dataset. Finally, we provide a point-by-point response to the remaining issues raised by each reviewer.

A key issue raised by Reviewers 2 and 3 was the modest correlation between our spillover risk predictions and human seroprevalence. Reviewer 2 added that, given the modest correlation, it was unclear "what the results [of the manuscript] add over the referenced Mylne et al. paper." The Mylne et al. (2015) paper groups rodent and human LASV occurrences together in their training dataset and evaluate their model fit using out-of-bag presence and pseudoabsence data [2]. In contrast, we explicitly separate rodent and human data and use a more challenging method of validation that uses human arenavirus seroprevalence. Thus, the two methods are not really comparable in this way.

Admittedly, our spillover risk prediction explained only a small amount of variation in human seroprevalence. We believe that this weak relationship emphasizes the importance of human factors, LASV strain diversity, and alternative LASV reservoirs, that may also contribute to human spillover. In the Discussion, we now clearly describe how these additional factors might explain additional variation in LASV spillover:

- Discussion, line 507: This paragraph addresses potential human factors (housing, culture, etc.) that might affect the realized rate of LASV spillover, for a given level of risk (i.e., D_X) predicted by our model. In addition, we now mention that important variation in the level of LASV risk might also be lost because of the binary nature of our reservoir and pathogen models (i.e. LASV presence/absence in rodents, presence/absence of reservoir).
- Discussion, line 524: This paragraph discusses aspects of the phylogeography of LASV and the rodent reservoir that were not accounted for in the model.
- Discussion, line 539: This paragraph discusses other rodent reservoirs that could contribute to LASV spillover or modify the risk posed by the primary reservoir.

The benefit of our framework is that additional factors that might influence spillover risk (e.g., other rodents that host LASV) can be incorporated into the model. The extent to which different factors explain spillover risk in humans can then be assessed, and intervention strategies can be specifically designed around these risk factors.

* * *

SHARED COMMENTS OR CHANGES TO THE MODEL AND DATASETS

* * *

- Reviewers 1 and 2 both requested more details of the model fit. We've added more details of the generalized linear model to the Appendix (Section 3). We now explain that the quasi-binomial framework is based on a binomial regression with an additional scaling factor that multiplies the variance (i.e., the dispersion parameter). Though both types of regression models give the same coefficient estimates and predictions, the quasi-binomial regression accounts for increased variability in the residuals when testing for coefficients that are significantly different than zero.

  To help motivate the use of a quasi-binomial regression, we've added a plot of the deviance residuals for a binomial regression (S1 Appendix, Fig 5). This plot shows 1) that the residuals are homoskedastic with respect to the fitted values, and 2) the residuals are overdispersed.

  The quasi-binomial is not based on an explicit probability distribution, so it is not appropriate to discuss likelihood based measures of fit such as deviance. Instead, we report the null and residual deviance, as well as the quantiles of residual deviance, for the binomial regression (S1 Appendix, Table 1). We do, however, report the results of hypothesis testing on the coefficients in the quasi-binomial framework (Appendix, Table 2). In addition, we now mention the estimate of the dispersion parameter in the main text (line 381).

- Reviewers 1 and 2 both commented on the omission of LASV-induced mortality (i.e., LASV virulence) in the model. Because of this, we've incorporated a virulence term μ into the human SIRS model. Humans still transition out of the LASV infection class at rate γ, however, only a fraction $\gamma(1 - \mu)$ transition into the recovered class and the remaining fraction $\gamma\mu$ die of LASV infection. The incorporation of this death rate increases the LASV case rate estimates by a factor $\frac{1}{1-\mu}$ uniformly across all of West Africa. For the 2% mortality estimated in McCormick et al. (1987), the increase is by a factor of 1.02.

- In the first draft we assumed a 50 year lifespan for humans in West Africa. Per Reviewer 2's suggestion, we now use country-specific estimates of lifespan from WorldBank to calculate the number of LASV cases in West Africa [3]. Specifically, the value of d, the natural death rate, within a given pixel is determined by the 2018 estimated lifespan L ($d = \frac{1}{L}$) for the country in which that pixel falls. 2018 was chosen because it was the most recent lifespan estimate available.

- Reviewers 1 and 3 both called into question the utility of the estimates derived from the spatially uniform LASV infection model. We intended for this model to be an intuitive, simple estimate of the LASV case rate from which the reader could better understand how the spatial model's estimates were derived. We now agree with the reviewers that the null model does not add to the paper, and have removed it from the manuscript.

- Reviewers 2 and 3 both suggested that a data-table be added to the manuscript to help the reader understand the spatial and temporal range of the dataset. We've added three tables to the main text that summarize the temporal and spatial range of each dataset. Table 1 summarizes the rodent capture dataset, Table 2 summarizes the rodent LASV dataset, and Table 3 summarizes the human LASV seroprevalence dataset.

  For the human data-table (Table 3), we initially tried versions that featured one study site per row for all published sources. However, this led to a 2+ page table that was redundant, and not as useful

for computation, as the CSV file that contains the dataset on the Github repository. Thus, we chose to summarize the data in a form where each row describes a single reference. Both of the rodent datasets include entries from GenBank and the literature. Because the rodent data within each pixel is aggregated together into a binary response (LASV +/-), it is more complicated to organize around individual references. We instead summarize the rodent data so that each row is a summary over a country in West Africa.

- In addition to incorporating tables into the manuscript, we've added a Microsoft Excel workbook version of the data to the github repository. This Workbook provides an easy interface for readers that want to view specific details of the dataset. For each human serosurvey, we include a "notes" column entry that provides any information from the article on how survey sites were chosen, as well as how individuals were chosen within survey sites.

  The LASV workbook contains three spreadsheets: the first shows the raw dataset consisting of literature sources that were examined, the second shows a cleaned dataset of literature sources, and the third shows the GenBank data. In the cleaned literature spreadsheet, we removed the entries that were not eligible to be used in training the model. These were survey data, for example, with missing latitude / longitude, LASV reservoirs other than *Mastomys natalensis*, or entries with incomplete human survey data, that were automatically removed in the data processing script of the pathogen layer.

  Per Reviewer 1, we've also restructured some of the columns of the dataset to better communicate the diagnostic methods. New columns "Virus Diagnostic Method" and "Ab Diagnostic Method" more clearly show which diagnostic technique was used in each rodent or human survey.

- Our counts of rodent LASV presence and absence pixels, as well as our counts of human serosurvey sites, have changed. These changes, in turn, are a result of 1) a minor error in the data processing script, 2) updated GPS coordinates in a few sites, and 3) adjustments made to human serosurvey data.

  1. In the pathogen layer of our model, a site is LASV negative if at least 5 *M. natalensis* are tested and all LASV antibody and virus-detection tests are negative, and positive if LASV virus is detected by isolation or PCR. Because arenaviruses cross-react serologically, the presence of LASV antibodies only imply past arenavirus infection. Therefore, we meant to omit any pixels that only contain seropositive rodents without any corresponding Lassa virus detection. The five sites for which antibodies, but not virus, had been reported were accidentally being counted as absences. This error has been fixed, resulting in five fewer rodent absences (previously 36, now 31). We are grateful for Reviewer 1's comment that allowed this error to be discovered.
  2. We updated some village GPS coordinates. Previously, if a site's GPS coordinates were not given in the primary manuscript, the coordinates were obtained through a google-earth search of the site name. This method was later found to be unreliable because multiple locations could have the same name. Sites for which latitude/longitude were obtained in this way have been updated with help from an established data-base from Fichet-Calvet, Rogers (2009). The updated coordinates affected three sites. Specifically, three rodent LASV-positive study-locations now overlap with other LASV positive sites, resulting in a net reduction of three positive sites in the pathogen layer of the model (previously 38, now 35).
  3. We made a few refinements to the human serosurvey dataset. Because reviewers 2 and 3 requested more details on human serosurvey design, we omitted a serosurvey mentioned in Monath, Mertens (1973) that provided no details on survey-design. The survey is still in the "raw" spreadsheet

in the LASV dataset, but is no longer used in the GLM. Secondly, we combined three entries from Sogoba, Rosenke (2016) that were survey results of the LASV IgM antibody with LASV IgG survey data from the same three locations in the same study. We also omitted the survey data from Akoua (2006) which aggregated surveys from multiple locations in a district. Finally, a few entries from Yalley-Ogunro were paired surveys of the same site separated by 2 years. These paired surveys were combined. These changes reduced the number of serosurveys that are used in our analysis from 101 to 94.

- We've changed the data filtering process that is applied to the rodent capture data. As a result, the numbers of *M. natalensis* presence and background pixels have changed. We now require each *M. natalensis* record in the dataset to have an associated article that documents the capture and the year that the rodent was captured. This criterion omits captures that were referenced as "personal communication" to E.F.C.

  We added three criteria for an entry in the original GBIF rodent capture-data to be used in the reservoir layer. The first criterion was motivated by concerns that reviewers 2 and 3 had regarding the time-frame over which the datasets were collected. Now, we only include GBIF entries that document a capture in the year 1977 or later. This year was chosen so that our background capture data are no earlier than the time-span of the presence data, which spans 1977 – 2017. Secondly, because *M. natalensis* and *M. erythroleucus* are difficult to distinguish based on morphology alone, we now exclude all GBIF entries that are within the genus Mastomys from the background data. Thirdly, we added a requirement that each background record is based off of a preserved specimen or tissue sample, thus omitting rodents identified through human observation or photographs. These changes reduced the set of background points from 897 to 252. Despite this large reduction in background points, the dataset remains evenly distributed across the study region and the reservoir layer of the model still robustly converges.

- Changes that were made to the LASV dataset did not have a substantial impact on the learned relationships of the pathogen layer. However, the changes to the rodent capture dataset did influence the predictors that the reservoir layer learned to associate with the presence of *M. natalensis.* Now, *M. natalensis* is predicted to be present in areas with strong seasonal vegetation patterns in addition to specific levels of precipitation. We've updated parts of the manuscript that describe these relationships (Section 2 of the S1 Appendix).

* * *

POINT BY POINT RESPONSE TO INDIVIDUAL REVIEWS

* * *

REVIEWER 1

* * *

Comment: 1. On line 436 of the Discussion, the authors raise the issue of contrasting dynamics of multiple genotypes of LASV. This is a critically important consideration for LASV and also deserves consideration from the perspective of the reservoir host, since previous work has shown that that different sub-species of M. natalensis carry genetically distinct different arenaviruses all closely related to LASV but

with varying capacity for infecting humans (see Gryseels et al. 2017 Plos Pathogens). I would imagine that human serological responses to a number of these arenaviruses may be impossible to differentiate, but modeling their dynamics collectively amongst a variety of hosts is not correct. At the very least, the authors need to include some discussion of these considerations. Better, I would like to see the sub-species of M. natalensis reported or at least summarized so that we can better understand the accuracy of these risk layers that treat all M. natalensis as equal.

Response: We agree and have incorporated this information in the manuscript:

- We've used this line of research to better motivate our choice of study region in the Data section (Line 76). Specifically, our study region is essentially the geographic distribution of the A-I *M. natalensis* clade [4].
- We expanded the Discussion section to include a paragraph on the interplay of host-virus phylogeography in arenaviruses (line 524) with references to Colangelo et al. (2013) and Gryseels et al. (2017). The text also emphasizes that our forecast should be interpreted with caution in eastern Nigeria, as this is the transition zone into the A-II clade. Because our study area is focused on that region occupied by the A-I clade, we do not include clade information in our dataset.

* * *

Comment: 2. The authors highlight that their spatial 'risk' model is likely to be more accurate than previous because it is limited to reservoir data only, and rodents are much more restricted in their movements than humans. That obviously could explain why their risk predictions only show a weak association with regional human seroprevalence (e.g. the humans are moving around), but it is important to note too that there is vast uncertainty in the regions where essentially no surveillance has taken place, as rodents are likely to be sampled (and in particular tested for LASV) in regions where humans are known to have been previously infected. In particular, the authors include Togo and Benin in their risk map, but it appears that neither country has reported any sero-surveillance of rodents for LASV, which does not mean that these regions are low risk but rather that we don't know anything about [these countries].

Response: It is true that Togo and Benin are not represented in the dataset that is fed into the model. We now mention that these countries are in need of more rodent and human sampling effort (line 450). However, an important contribution of the risk sub-models is to learn relationships between the distribution of LASV-infected *M. natalensis* and the environment where data *are* available. These learned relationships are then extrapolated to new regions within a relevant study area. Furthermore, the predictions of our model in these poorly sampled areas are testable and can be used to validate our model or identify areas where predictions must be improved.

* * *

Comment: 3. The second layer pixel map for whether a region is deemed to be LASV positive vs. negative is derived from PCR tests of M. natalensis captures for LASV. Intriguingly, the authors choose to represent the region as LASV negative if either serology or PCR-based results in rodents show no evidence of LASV infection. This is reasonable, given that a seronegative result shows no back history of exposure. However, the authors only designate a region as LASV positive if there is PCR-based detection of LASV infection in M. natalensis, and no mention is made of what is done if there is not PCR-based evidence but if the rodent population showed seropositivity. I am concerned that PCR-based detection is unlikely to pick up on seasonal shedding of virus (which we know is important for LASV – see Akhmetzhanov et al.

2019 Phil Trans Roy Soc), but the metadata posted to github appear to not show the seasonality of infection. In fact, some of the diagnostic methods in the data are listed as “various” which provides no information at all. I would like to understand if there were positive serological data in the rodents that were excluded and why.

Response: Because many arenaviruses cross-react with LASV antibodies, serological (i.e., IFA) tests are not LASV specific. Rodent sampling work of Olayemi, Oyeyiola (2018) in Nigeria demonstrates some of the ambiguity that can arise. In one of their study sites, Mayo Ranewo, 16/31 *M. natalensis* tested positive for arenavirus IgG. However, 0 tested positive for LASV by way of PCR and 1/31 tested positive for a Mobala-like virus [5]. The seropositive rodents could have been exposed to Mobala virus, LASV, or possibly another arenavirus. Because there is no other source that indicated LASV is present in that pixel, the LASV status of the pixel is deemed ambiguous (instead of LASV present) and omitted from the dataset. While we agree that excluding serologically positive rodent data could result in false negatives, including studies that only indicate arenavirus presence through serological tests in a pixel could just as easily introduce false positives for Lassa virus. This is especially true in the transition zone between matrilineages A-I and A-II (i.e., where Olayemi, Oyeyiola (2018) takes place.

This choice does not significantly change the dataset or the model. Our data-processing script omits five pixels that each contain one study that documents arenavirus seropositive rodents, but for which Lassa virus has not been detected (Table 1).

Table 1: **Sites at which seropositive rodents have been found, without any explicit detection of Lassa virus.**

Ab+	Ab Tested	Location	Source
1	33	Saorou, Guinea	Fichet-Calvet, Becker-Ziaja (2014)
1	28	Banzana, Mali	Safronetz, Sogoba (2013)
16	31	Mayo Ranewo, Nigeria	Olayemi, Oyeyiola (2018)
1	99	Abagboro, Nigeria	Olayemi, Oyeyiola (2018)
5	24	Ngel Nyaki, Nigeria	Olayemi, Oyeyiola (2018)

Because of this comment, we’ve added an option in the code for the Pathogen Layer that allows these ambiguous pixels to be counted as LASV presences in the model pipeline. Counting these points as presences does not substantially change the model fit: 1) precipitation contingency is still the predominant predictor of LASV presence or absence, 2) The validation AUC is still relatively high (= 0.78), and 3) the significance of the resulting D_X layer in the human seroprevalence GLM remains strong (D_X coefficient p-value of 0.000245 when ambiguous points are counted as presences, vs p-value of 0.000154 when ambiguous points are omitted). We’ve added text to the Data section that describes the rationale behind omitting ambiguous pixels, the number of pixels that are omitted, and the fact that this choice does not dramatically alter the results (line 139).

* * *

Comment: 4. Point 3 leads me to also highlight the absence of incorporation of any signature of seasonality in infection risk in this paper – risk is measured exclusively as a spatial effect under equilibrium assumptions which just is not accurate. We know full well that there is a risk season for Lassa which takes place in the Dec-Jan months that correspond to the dry season and the rodent breeding season and this is barely even discussed, let alone explored in detail.

Response: We agree with the reviewer that temporal fluctuations in the spillover rate occur and are important. However, we also believe there is a spatial patterning of LASV spillover that is dictated by the

presence of rodent reservoirs of LASV, as well as the distribution of LASV in those rodents. The rodent reservoirs and the virus are, in turn, patterned by relatively stable features (e.g., precipitation patterns, vegetation patterns, etc.) of the environment. It is this relatively static spatial signal that our model is constructed to learn. This is appropriate because these risk outputs are being used to predict human serosurveys that potentially track the average rate of LASV spillover across decades (i.e., the longevity of IgG antibodies in humans) [6, 7]. Moreover, datasets that describe the temporal variation of LASV infection in humans or rodents are extremely limited across West Africa.

Because of this comment, we've broadened the discussion to emphasize several "steady-state" assumptions that were needed to calculate the rate of LASV spillover into humans. We now include reasons why the average annual LASV spillover rate is likely changing on a decades-long time-scale (paragraph at line 550), and in another paragraph, discuss several factors that are believed to drive the seasonal changes of LASV spillover (paragraph at line 561).

* * *

Comment: 5. In addition, building on point 4, the authors use a somewhat strange approach to predicting seroprevalence spatially, assuming the absence of spatial fadeouts of human infection which are almost a certainty for a rare zoonosis with a distinctive seasonality. It would be much more compelling if they could make this a catalytic model predicting age-seroprevalence (e.g. Muench 1959) and a constant hazard of seroconverting across an individual's lifespan. I did not go through all the reported human data in detail, but at least a subset of it appears to have age data associated. This paper would be much stronger if the authors made an attempt at this.

Response: We agree that our model is not designed to detect temporal changes in the rate of LASV spillover, but instead detects the average historical spillover rate of LASV.

Analyzing human seroprevalence across age groups would be a fascinating research direction for future work. It might be interesting, for example, to look for spatial fade-outs in the age-seroprevalence human data, and relate these to aspects of the reservoir or pathogen models. However, this direction would be limited to studies that report the age-seroprevalence relationship for each individual village sampled. Unfortunately, many of these studies do not discuss the age-seroprevalence relationship or, if they do, aggregate this relationship over sample locations. As before, it is likely that sparse data would make finding this relationship even more difficult.

* * *

Comment: 6. Additionally, the authors report estimated seroprevalence and incidence for assumptions of both SIRS and SIR. There are certainly compelling studies out there suggesting that human Ab immunity to LASV can be lifelong (e.g. Bausch et al. 2013 ASTMH) which contradicts the SIRS assumptions and provides a more reasonable (i.e. lower) estimate of cases. I would like to at least see the emphasis on SIRS justified...

Response: Our goal was to provide a broad range for the possible number of LASV cases and highlight the importance of understanding the rate of seroreversion. Anecdotal case-studies do indeed suggest that LASV antibodies can remain in humans for decades [6]. However, there is also research that shows that: 1) Because LASV immunity is at least in-part cell-mediated, protection against LASV does not necessarily correlate with antibody titer, 2) protection against LASV reinfection wanes with time in nonhuman primate studies [8], and 3) Antibody titers wane with time [7]. We believe this is enough evidence to at

least investigate the possibility of waning immunity. We've added these points to the Methods (line 302) to help motivate the need for estimates that include the possibility of LASV reinfection.

We've softened the emphasis on the estimates that include seroreversion in the Results section. Specifically, we've modified the Results to first focus entirely on our model predictions in the case where LASV immunity is lifelong in humans (paragraphs at lines 387 and 399). In the following paragraph at line 408, we describe the effect of seroreversion on our case estimates.

* * *

Comment: 6. (continued) ...and it would be useful to see some comparison of a boosting model where low Ab titers wane but can be boosted upon re-exposure. Given that humans are not modeled as infectious, it should not change the dynamics but it might fit the data better...

Response: Given that our model is evaluated at steady-state, this change is mathematically equivalent to reducing the rate λ at which seroreversion occurs. The results we provide already describe the LASV case rate estimates for the range spanning no seroreversion (lifelong LASV immunity, $\lambda = 0$) to those levels of seroreversion measured in McCormick et al. (1987) (average duration of LASV immunity is 15.6 years, $\lambda = 0.064$ yr^{-1}).

* * *

Comment: 6. (continued) ...Which leads me to ask—how well do these models fit the data? I would love to see some statistical output of that line's fit to the data in Figure 4.

Response: Addressed at the top of this document.

1.1 General Comments

Comment: Author Summary: SARS-2 coronavirus is incorrect terminology. It is SARS-CoV-2 and coronavirus is already represented in the middle of the term

Response: Fixed.

* * *

Comment: Line 44: It is important to caution that these assumptions probably don't hold for all reservoir species (e.g. bats are highly mobile)

Response: We modified the sentence to read (line 42): "If the dispersal ability of the reservoir is small, training the risk layer on reservoir cases alone helps the model avoid these biases when learning the spatial variation of spillover risk."

* * *

Comment: Line 49: I know that ambisense viruses are still classed within the negative sense viruses for taxonomic convenience but it is a bit strange here to see that you class Lassa as both negative sense and ambisense. Suggestion to just stick to ambisense here.

Response: Changed.

* * *

Comment: Line 91-92: What about sub-species? This is super important to Lassa virus dynamics – see comments above

Response: We've added text to the Data section that describes how different clades of *M. natalensis* likely differ in their ability to transmit LASV (line 76). We then use this fact to better motivate limiting the study region to West Africa, the range of the A-I clade.

* * *

Comment: Line 114: PCR tests really need a seasonal component to pull off this Lassa positive/negative risk map. Why are serological studies not included here but are included in evaluation of Lassa negative regions? See comments above.

Response: Addressed above.

* * *

Comment: Line 254: Spillover risk is modeled as FS, where F is the regional force of infection from the reservoir and S is the human population density, which assumes this to be a density-dependent transmission process. Can you justify this assumption? This is what leads you to conclude that Nigeria is at the highest risk (they have the most people), but that does not necessarily mean that there will be a higher number of human-rodent contacts. Unless rodent density increases with human density (which it may), it seems like a single rodent would have a fairly limited movement range and likely a discrete number of likely human contacts.

Response: The density-dependent form FS is valid, for example, if changes in the density of susceptible humans (S) at the population level result from changes in humans per dwelling. In this scenario, a fixed force of infection F in a household (i.e., due to the presence of rodent droppings, urine, or direct interactions) would result in more human infections as the number of human inhabitants increased. We've added text to the Discussion (Line 436) that points out this assumption.

Moving away from this assumption would require estimating how human-rodent interaction changes with human population size. This, in turn, could be influenced by community structure, local customs, and income. We agree that altering this density-dependent assumption would be interesting, but we do not believe that it is possible with the data that are available.

* * *

Comment: Line 258: This lack of mortality effect is a strong assumption in a disease that has known mortality. I accept the need to model it this way, but how might consideration of this change results?

Response: The model now includes mortality due to LASV infection. Addressed at the top of this document.

* * *

Comment: Figure 1: It seems that you can't say anything really about the risk of LASV spillover in Mauritania, Burkina Faso, Senegal, Niger, Benin, or Togo since there are no rodent infection data to actually evaluate.

Response: We believe that our machine learning model learned relationships between the presence of LASV in rodents and environmental variables. These learned relationships apply outside of the point-wise locations for which LASV rodent infection data are available. However, we now point out that these areas are need of more LASV surveillance in the Discussion (line 450). We view these predictions in areas lacking data as unique and testable predictions of our model that expose it to rigorous testing as data becomes available from these regions.

* * *

Comment: Figure 3: As in above, this figure highlights that the "risk map" of potential spillover for Lassa is really just an absence of surveillance. The regions where infected *Mastomys natalensis* have been previously tested pop up and those regions where they have not do not.

Response: The absence of LASV data in rodents from these regions does not necessarily lead to predictions of low risk. Instead, predictions of low risk indicate that the region's predictors have more in common with surveyed pixels that are LASV-absent (given our definition) than pixels in which LASV was detected.

* * *

Comment: Figure 4: Can you provide some statistical output on the findings in this figure?

Response: Addressed at the top of this document.

* * *

Comment: Line 345: This is extrapolating way too far – I would advocate to restrict the projections to the more conservative spatial estimates. It seems like this is reported here just to have a dramatic number.

Response: We've removed the null model from the manuscript.

* * *

REVIEWER 2

* * *

2.1 Major comments

Comment: This paper appears to be an important next step in a fairly sparse literature (at least from my cursory searching). However, right now it is predominantly an eloquent presentation of a method (with some novel ideas) but with only a cursory consideration for the biological implications. I am also a little confused by the methods: combining the two layers by multiplying them, then using the binary classifier score as a predictor for a continuous outcome in the GLM seems like a weird pipeline. Given that the binary classification score (from the first two layers) appears to have little explanatory power in the GLM, I am not sure what the results add over the referenced Mylne et al. paper. I think these methodological concerns need to be clarified and the implications of the results (rather than just the unrealized potential of the method) made clearer if this is to warrant publication in PLoS Comp Biol.

Response: What our manuscript adds over the Mylne et al. paper is addressed at the top of this document. The reviewer's concerns about the binary classification setup are addressed in subsequent comments below.

* * *

Comment: The authors claim to be modelling spillover risk, but I am not convinced that is what their model is predicting. I am probably missing something crucial, but it seems odd to train the model on binary data (presence/absence of LASV or M. natalensis) and to then use the predictions as a quantitative estimate of spillover risk. The model may predict a high classification score for presence of LASV infected

rodents in a pixel, but does that necessarily mean that it is predicting higher LASV prevalence in the reservoir population? Is it distinguishing between a small rodent population with low prevalence and a large population with high prevalence? It seems like those data points are weighted the same here.

Response: We fully agree with the reviewer that a model trained on rodent abundance and LASV prevalence would give a more mechanistic prediction of human spillover risk. However, both LASV prevalence, as well as the density of rodents inside of houses (where human contact is likely), vary substantially by season [9]. Because of this variability, and because of the sparsity of temporal studies on LASV and the LASV reservoir, prevalence or density data across West Africa will be heavily influenced by the season in which such data was collected. Furthermore, estimates of rodent density (i.e. trap success) are influenced by trapping methods (e.g., trap density, bait, trap nights), which differ between researchers. Because of these shortcomings of the available data, we simplified our reservoir datasets into binary classifications that are more robust to these sources of variability. This step of data-processing is now more clearly motivated in the Data section (line 133) and is also mentioned in the Discussion as a possible cause for the weak correlation with human seroprevalence (line 508).

The product of these binary classifiers should still correlate with the magnitude of spillover although the correlation will be weaker. First, predictions in the spillover model convey confidence in a classification (i.e. $D_X \approx 0$ implies high confidence that a site is missing the rodent or the virus; $D_X \approx 1$ implies high confidence that the site contains both rodent and virus) that likely varies with the extent of LASV spillover into humans. Secondly, because of the sampling error that is inherent in any random survey, locations in which at least one LASV-positive rodent was found will tend to be those that have a higher LASV prevalence than those in which all rodents tested were LASV-negative. If this is the case, the binary data of the pathogen-layer of the model will correlate with prevalence of LASV in rodents.

As part of ongoing research, we are currently working to collect more detailed estimates of rodent abundance and LASV prevalence in Sierra Leone and Guinea. Once systematic reservoir datasets are obtained, we will update the rodent and pathogen layers to explicitly describe rodent abundance and LASV prevalence.

* * *

Comment: Therefore, from what I understand, the method is only predicting the binary presence or absence of at least 1 LASV infected rodent which is what Mylne et al. did (they call it suitability index rather than spillover risk). If so, what do the authors think the GLM would look like if Mylne et al's suitability index were used instead?

Response: We've tried to clarify that the focus of our manuscript is to understand the amount of spatial variation in human arenavirus seroprevalence that is attributable to aspects of the reservoir (addressed at top of document). The suitability index proposed by Mylne et al. is informed by LASV infection data from rodents and humans. As such, it conflates the LASV risk posed by rodents with human behaviors that may modulate the local risk. Our results suggest that LASV risk from infected *M. natalensis* does not explain much of the variation, which is something that the Mylne et al. (2015) framework is not designed to investigate.

* * *

Comment: The GLM clearly does work given that it explains some variance and the slope is significant. However, if the predictor is only binary, then isn't the comparison to magnitude of human seroprevalence

somewhat flawed? For example, a classifier for "absence, low LASV prevalence in rodents, high prevalence LASV prevalence in rodents" would make more sense if the aim is to predict magnitude of risk. Is a lot of quantitative information being thrown away converting to a binary prediction, and is this why the GLM appears to explain such little variance? Maybe I am trying to be too mechanistic, but I think this would be helped if the methods and results clarified why this approach makes sense.

Response: Addressed above.

* * *

Comment: Figure 2 and associated methods: I have been trying to intuit why simply multiplying the likelihoods makes sense, but something feels off. The D_M layer is predicting the likelihood of M. natalensis presence given the environmental variables, whereas the D_L layer is predicting the likelihood of LASV circulation given the environmental variables and the presence of M. natalensis. I would recommend adding some text to explain the intuition behind this. A bit of a half-baked thought, is it related to the conditional probability P(LASV | rodents) * P(rodents)? If so, is multiplying the likelihoods the right approach?

Response: Correct. The pathogen layer of the model is trained on data that describe tests of *M. natalensis* for LASV. Because the layer is only trained on pixels in which the rodent is present, the probability that it returns is necessarily conditioned on *M. natalensis* being present in a pixel. Letting Mn denote the event that *M. natalensis* is present in a pixel, and letting LASV denote the event that LASV is present, we have

$$D_X = Pr(\mathrm{Mn} \cap \mathrm{LASV}) = Pr(\mathrm{LASV}|\mathrm{Mn})Pr(\mathrm{Mn}) = D_L\, D_M.$$

We made it clearer that D_L describes a conditional probability (line 191): "The layers of risk, in turn, are described by: 1) D_M, a classification score indicating the likelihood that a pixel contains the primary rodent reservoir, *M. natalensis*, and 2) D_L, a score indicating the likelihood that LASV circulates within the *M. natalensis* population, conditioned on the rodent being present."

* * *

Comment: Discussion of the implications: does this new denominator change our understanding of the Lassa fever clinical spectrum and epidemiology? For example, if the IFR is 2%, then 3 million infections implies 60,000 deaths. Is this consistent with underreporting of LF-associated deaths, or does it imply that the IFR is set too high?

Response: Because we do not know how many unreported deaths from LASV-infection occur each year, it is difficult to say. Line 466 of the discussion mentions that our estimates exceed those reported by the Nigerian CDC.

* * *

Comment: Figure 4 is pretty underwhelming: it leaves the reader feeling unconvinced that the modelled relationship is real or useful. The plot should at least have confidence intervals on the model line. Also is this from the weighted or unweighted version?

Response: We've added confidence intervals to the figure. The modest fit is addressed at the top of this document.

Clarification on the usage of "weights": the GLM used in the regression weights each survey by the number of individuals tested. In the original manuscript, we also reported the correlation between the model's

predictions and the actual human serosurveys. Two correlation measures were calculated: one with the number of individuals in each survey included as weights, and one without weighting. We've removed mention of these correlation measures in the manuscript.

* * *

Comment: ...relating to Figure 5 (see minor comments), is this actually doing much better than assigning each pixel the mean seroprevalence?

Response: Yes, the quasi-binomial GLM output shows that the D_X coefficient is significantly different than zero. Importantly, the GLM tests this hypothesis while accounting for overdispersion (i.e. inflated variance) of the data. A dispersion parameter of $\phi > 1$ multiplies the estimates of standard error on coefficient estimates by a factor $\sqrt{\phi}$, which in turn lessens the probability of a Type I errors (i.e., falsely assigning significance to a predictor because of overdispersion). Even with this correction, the coefficient of D_X was deemed significantly different than 0. This implies that the model with the coefficient is better than the model with constant seroprevalence.

* * *

Comment: The discussion is well written and raises a lot of interesting points. However, it does not discuss the results from the D_M and D_L layers at all. For example, it is not clear how to interpret the result that maximum precipitation and precipitation contingency are the 2 most important predictors in both layers. Are the key environmental predictors the same or different to previous work, and are there any novel biological insights there? Line 467 hints at this, but I didn't feel like the discussion clarified what these insights are. There is some text in the supplement, so this should at least be referred to.

Response: We added a paragraph to the discussion that puts into context the environmental features that influenced the occurrence of the reservoir and pathogen (Line 491).

2.2 Minor comments

Comment: The omission of a time component in the model seems important. The reader doesn't know when e.g., the rodents were captured relative to e.g., when seroprevalence was measured.

Response: Addressed at the top of this document.

* * *

Comment: Is there some evidence to support the assumption of steady-state seroprevalence rather than increasing seroprevalence over time? Are there any longitudinal or repeated cross-sectional studies?

Response: There is little information on how LASV spillover or human seroprevalence changes with time. Yalley-Ogunro, Frame (1984) describes longitudinal serosurveys that occurred 2 years apart and found similar seroprevalence in both of the villages sampled. Aside from that, it is well-known that LASV spillover regularly occurs in areas of Guinea, Sierra Leone, and Nigeria. We now explicitly mention how LASV spillover may violate this steady-state assumption in the Discussion paragraph at line 550.

* * *

Comment: I think there is an opportunity to further test the sensitivity of the D_M layer. You have an independent dataset for true positive M. natalensis presence: the data used to train the D_L layer. Does the

D_M layer accurately predict the presence of M. natalensis in those locations? This might be a useful small supplementary analysis to support the accuracy of the D_M layer.

Response: Locations at which *M. natalensis* were identified with genetic methods and tested for LASV are already included in the reservoir dataset. However, we do perform the type of validation the reviewer suggests. Specifically, the training dataset for each boosted classification model is sampled from the full dataset with replacement. As a result, approximately 1/3 of the full dataset is left out of each training iteration and used for testing the fit of the resulting model. This is the out-of-bag cross-validation described on Line 242.

* * *

Comment: The AUC for the same predictors (absence/presence of M. natalensis or Lassa) looks almost identical to those in Table 2 of Mylne et al. Does this mean anything, given that the top environmental predictors are the different?

Response: These similar AUC metrics indicate that each of the model layers are performing similarly in their ability to discriminate between presences and absences in their respective datasets. However, comparing the predictors is made difficult by two key differences between our approach, and the approach of Mylne et al. (2015). First, the reservoir submodel in Mylne et al. (2015) is trained on *M. natalensis* occurrences from all of Africa. We chose to crop the *M. natalensis* presences (and background rodent presences) to West Africa in order to focus on the A-I clade that is believed to contribute most to LASV spillover (discussed in the revised manuscript at line 78). Secondly, in Mylne et al. (2015), the *M. natalensis* model output is fed into the LASV suitability model as a predictor. This, in turn, changes the importance of predictors of LASV that contributed to the presence of *M. natalensis*.

* * *

Comment: Line 41: It isn't clear to me how the issue of human seroprevalence not coinciding spatially with location of infection is resolved here. When comparing the model predictions to observed seroprevalence in the GLM, is this issue not being re-introduced anyway? Consider clarifying.

Response: We've added text to clarify this point. The spatial variation of spillover risk (D_X) that our model learns is based on attributes of *M. natalensis* only, and therefore is not influenced by human movement. It is true that human seroprevalence is used to link D_X to the LASV case-rate in humans. However, the serosurveys used typically involve many individuals and are therefore less likely to be influenced by human movements. We've modified the text in question to read (line 39): "First, in the case of LF, due to modern transportation and the longevity of Lassa virus antibodies in humans, a general concern is that the reported location of individual cases of human disease or Lassa virus antibody detection is not the site at which the infection occurred [6, 10, 11]. If the dispersal ability of the reservoir is small, training the risk layer on reservoir cases alone helps the model avoid these biases when learning the spatial variation of spillover risk. Secondly, in our framework, human seroprevalence estimates provide an ultimate test of the risk layer's ability to correlate with spatial variation in the cumulative human exposure to the pathogen. The seroprevalence data, in turn, stem from population-based surveys at a site and are therefore much less likely to be influenced by the movement of individuals."

* * *

Comment: Line 70+ The date range for the animal and LASV data is not mentioned, but it is for the human seroprevalence data. Would be good to see this to understand how comparable the dates are.

Response: Addressed at the top of this document.

* * *

Comment: Line 70+: Similarly, the survey data are not described in much detail. I understand that there are so many sources that this would take up too much text, but a supplementary table, particularly for the human seroprevalence data, describing sample sizes, demography, protocol etc might be useful.

Response: Addressed at the top of this document.

* * *

Comment: Line 121: The text suggests that the human seroprevalence data are surveys of all arenaviruses antibodies, not specifically Lassa. Perhaps this is obvious to someone with experience with these pathogens, but is there a reason why the included studies were not LASV specific? Are the assays highly cross-reactive or are there no other arenaviruses that infect humans? This should be clarified.

Response: We agree and have clarified that arenavirus antibodies cross-react. As a result, a positive serology test only indicates past exposure to an arenavirus, and not necessarily LASV. In the revised text, this is mentioned in the Data section on LASV surveys in rodents (line 139), as well as the Data section on human seroprevalence (line 159).

* * *

Comment: Line 130: I understand the decision to only include land types that have been consistent for 20 years from a methodological perspective, but does this not ignore a major source of spillover risk – increased activity at the animal-human interface? It's not my area so I may be wrong, but if this is important, its implications and limitations should be discussed in the main text. From the abstract of the cited Gibb et al. paper: "Although the recent increase in LF case reports is likely due to improved surveillance, recent studies suggest that future socio-ecological changes in West Africa may drive increases in LF burden". Line 444 mentions time but not in the context of land type. Perhaps mention this here.

Response: This is a fair point and we've added it to the discussion (line 554) in a paragraph that summarizes the steady-state assumptions on which our predictions are based.

* * *

Comment: Line 177: Is a Bonferroni correction for multiple comparisons appropriate here? Probably not given that this is just an arbitrary pre-filtering step, but something to consider.

Response: We do not feel that the Bonferroni correction is necessary. We are not trying to test whether a group of predictors is simultaneously significantly associated with positive or negative pixels. Rather, this step is meant to be an automated pre-filtering step to discard predictors that are unrelated to the response variable.

* * *

Comment: Line 207: Maybe I'm being slow, but I don't follow the logic for how false negatives make the classifier conservative. Please clarify.

Response: We meant that *M. natalensis* may be present in pixels that do not contain any records of the rodent being captured. We agree that this sentence was confusing and have removed it.

* * *

Comment: Line 209: Dumb question, but is this spatial distance or minimum distance across environmental variables?

Response: Spatial distance. We've clarified in line 249: "Specifically, for each test presence point, the pairwise distance sampling method chooses a test background point so that the minimum spatial distance between the training presences and test presence is similar to the minimum distance between the test background point and training presences [12]."

* * *

Comment: Line 258: Is it not a fairly easy addition to the model to assume some deaths? 2% mortality is a lot of deaths when you're predicting millions of infections! How would this relate to how we understand LF epidemiology now? Though I understand if this would mess up eq 2-4.

Response: Mortality due to LASV infection has been incorporated into the estimates. Addressed at the top of this document.

* * *

Comment: Line 266: is there evidence that arenavirus seroprevalence is at steady state or is this an assumption?

Response: This is an assumption. We changed this text (line 314) to "We assume that, within each pixel, the dynamical system given by System 1 is at steady-state. Consequently, the net rate of mortality is equal to the constant birth rate b, and each of the classes S, I, and R are not changing with time."

* * *

Comment: Line 280: Refers to the data section, but there is no information on the WorldPop data there. Is this text missing or is the reference erroneous? (I think it should refer to S1 Appendix).

Response: This reference was from a previous draft. Fixed.

* * *

Comment: Line 282: Assumed a mean lifespan of 50 years for all locations. Why not use country specific estimates?

Response: We've incorporated this change in the model. Addressed at the top of this document.

* * *

Comment: Line 281: The equations are correct, but I couldn't get my head around the idea of there being a constant "steady-state population size" if the real population size is growing (ie. when b > dN). I think I see now that this is a possible equilibrium because i) the incidence rate does not depend on the I compartment (as in a textbook example) and ii) crucially, the birth rate b is assumed to be not density dependent. I couldn't understand how the incidence of new infections could be constant while the population is growing and the proportion in each compartment stays the same. But I think it works because b just feeds in a constant number of new susceptibles (rather than an increasing number if the term were bN, which I think would be more standard). Nothing to be done if this is a trivial realization, but some text to explain the intuition for why this steady state exists and the rational for a flat birth rate might help a confused and pedantic reader like me!

Response: This is correct: our analyses assume that both the LASV infection process, as well as the population of each pixel in West Africa, are at steady-state. This assumption was necessary because for most locations our human seroprevalence data consist of a single snapshot in time.

We've added text to clarify this.

- Methods, line 280: "Throughout, we assume that the seroprevalence measures that were obtained from historical serosurveys describe LASV infection at steady-state (i.e., are unchanging in time)."
- Methods, line 286: "To facilitate steady-state analysis, we assume that new individuals are born in the pixel at a density-independent birth rate b."
- Methods, line 314: "We assume that, within each pixel, the dynamical system given by System (1) is at steady-state. Consequently, the net rate of mortality is equal to the constant birth rate b, and each of the classes S, I, and R are not changing with time."
- The Discussion paragraph at line 550 describes the various steady-state assumptions that are made in the model.

* * *

Comment: Line 292: At the moment, the authors present the immune waning estimate from McCormick et al. 1987 as an upper bound. Is there any evidence to suggest that waning might be even faster than the point estimate from McCormick 1987 (e.g., were there confidence intervals on lambda in that paper)? If so, this would be a useful scenario to present.

Response: No mention is made of the range of seroreversion rates in McCormick et al. (1987). The only reported estimate of seroversion is aggregated between villages: 6.4 reversions per 100 antibody positive individuals. This is the estimate we use for the parameter λ.

* * *

Comment: Line 319: missing space before the bracket.

Response: Fixed.

* * *

Comment: Figure 5: a histogram of residuals might be a useful subplot here. It's hard to assess how well the model is doing from this plot.

Response: We've added a plot of the deviance residuals vs fitted values, as well as a table that shows the quantiles of deviance residuals, to the S1 Appendix (Fig 5, Table 1). Addressed at the top of this document.

* * *

Comment: Line 376: This is a really key paragraph, but a bit more information on the surveys, for example, predominant housing type in particular outliers above/below the prediction line, could be useful. Was this information not available to include in the GLM here?

Response: The details on housing type are sparse in the serosurvey literature sources. Moreover, when these details are provided in a source, they are often aggregated to describe the range of housing sites. The intent of this manuscript is to lay the groundwork for future research into the additional human factors that modulate the spillover of LASV into humans.

* * *

Comment: I don't know this literature, but it looks like there may be a few relevant studies tackling a similar problem that are not discussed, e.g., https://besjournals.onlinelibrary.wiley.com/doi/full/10.1111/2041-210X.12549 which also provides quantitative spillover estimates

Response: We've added mention of Redding et al. (2016) in the Discussion (line 420, line 554).

2.3 Comments on code

Comment: Really nicely documented, well written and clean code. Great job. I ran everything line by line and found no obvious bugs! Only small comment is to not use reserved words (eg. data, grid) for user defined objects.

Response: We appreciate the kind words and have changed the variable names accordingly.

* * *

Comment: `Generate_Reservoir_Layer.r` L79: in the object 'classi.dat.rod', none of the pseudo-absence variables have countries or confidence. Just checking that this doesn't impact anything downstream.

Response: This is OK. Those columns do not influence the downstream processing.

* * *

REVIEWER 3

* * *

Comment: The paper would benefit from a framing that includes more about what we do and do not know about the distribution of human risk for Lassa, and what we would do differently if we did know these things to motivate the study. Currently, the introduction includes elements from the methods, results, and discussion and the paper would be easier to follow if the role of each section of the paper were more clearly delineated.

Response: We intend for this manuscript to focus on a machine-learning framework that can be applied to different wildlife pathogens with the capacity to emerge in humans. Lassa is an example pathogen to which the model is applied. Because of this, we believe that framing the introduction around spatial forecasts of emerging infectious diseases is appropriate. That said, we restructured the last paragraph of the Introduction (line 49) to better emphasize that 1) we do not know the extent to which the distribution of infected *M. natalensis* explains the distribution of seroprevalance in humans, and 2) we do not know the number of Lassa fever cases that occur in West Africa. In addition, we have removed sentences that describe the results of the manuscript.

3.1 Abstract

Comment: The abstract is very slim on results and offers few conclusions about what the next steps should be, given the new findings from this study. Consider cutting back on the background and rationale to make room for results and some discussion.

Response: Our manuscript is novel primarily because it allows the realized spillover risk in humans, as measured by human seropevalence, to be decomposed into different causal components (e.g., infected rodents, human behavior). We've rewritten the Abstract to emphasize this advance in spatial forecasts.

3.2 Introduction

Comment: Lines 3-4: Not all zoonotic pathogens circulate in a wildlife reservoir.

Response: We changed this sentence (line 3): "Approximately 60% of EIDs are caused by pathogens that normally circulate in wild or domestic animal reservoirs (i.e., zoonotic pathogens) [13]"

* * *

Comment: Lines 28-29: "As a result, the extent to which predicted risk explains the realized variation in human exposure to the pathogen is unclear." Might one way to evaluate this also be surveys to better understand exposures?

Response: The point we are making is that no other studies have capitalized on existing serosurvey data that describes the historical human exposure to LASV. Our study is unique in that we explicitly omit human LASV serosurvey from the training process. As a result, we can assess the extent to which the spatial variation in human seroprevalence is due to the spatial variation of infected *M. natalensis* rodents. New human serosurveys or infection data would certainly add to the dataset of realized spillover in humans, but we should also use existing serosurvey data that already exist.

* * *

Comment: The introduction would be more compelling if it focused on what is and is not known about risk to humans, a summary of what we already know about the frequency and distribution of human infections, and the gaps in our understanding of risk. Limits of the published models to estimate this risk are important to mention, but should also be placed within the context of measured seroprevalence. A clear statement of the study objective(s) would also be helpful for the reader.

Response: Addressed above in Reviewer 3's first comment.

3.3 Data

Comment: It would be useful to say something about when data were collected and the geographical extent they represent. It becomes clear as the paper goes along that the data are obtained through literature review, but it would be useful to have this stated upfront, perhaps with some of the methods used to identify the papers with data applicable to the analysis.

Response: We've changed the first sentence of the Data section to broadly mention the sources of the datasets, the time-range of the datasets, and the study region (Line 69). To help the reader understand the study-region, we've added a dashed blue line to Figure 1 that shows the study-region and moved the figure (and figure reference) closer to the start of the Data section.

We now include a detailed description of how the datasets were compiled (rodent capture data - line 92, LASV dataset - line 121). Because the criteria for inclusion are specific to each type of data, we save these details for the data subsections.

* * *

Comment: I'm not clear on why the description of where the data come from is not included in the section called methods.

Response: The dataset is extensive and comes from multiple sources. As such, we felt it was better to devote an entire section (renamed the "Data and Study Region" section) to describing the data.

* * *

Comment: The secondary data used in this study span many decades – it would be useful for the reader to have a table outlining the location and year(s) for the data used in the models.

Response: Addressed at the top of this document.

* * *

Comment: Line 94: The study area is unclear.

Response: Addressed above.

* * *

Comment: How are pixels defined?

Response: We've moved the pixel definition up in the text (line 83).

* * *

Comment: Lines 116-118: Can the authors provide some rationale for the definition of positive and negative pixels? Are there any limitations to the approach authors used to define positive and negative pixels?

Response: We've elaborated on the rationale behind the definitions of Lassa positive and Lassa negative sites. One primary limitation of converting sites into binary classification is that some information on the prevalence of LASV in *M. natalensis* is lost. We've clarified the definition and consequences in a couple places.

- Data section, line 133:"Because the prevalence of LASV in rodents varies seasonally [9], and because of the sparsity of time-series data that might otherwise allow the average LASV prevalence in rodents to be estimated, we used the collected data to broadly classify pixels into the categories "Lassa positive" or "Lassa negative"."
- The Discussion section includes text (line 508) that mentions how converting to binary LASV occurrence could lessen the correlation with human seroprevalence.

* * *

Comment: Lines 122-123: Regarding the requirement for individuals to be sampled randomly, would it not be sufficient for households within a village to be sampled at random, and individuals from those households included? Additional details about the literature reviews, including the papers that were excluded from the study would be useful for the reader to understand the process. (See comment about summary table above, which could also possibly include details about sampling methods.) Authors have restricted the serosurveys they included in the models, for good reason, to those that were conducted in some kind of random sample of the population. However, would be good to compare the known occurrences of infections with the resulting model predictions to see how they correspond.

Response: Our criterion for "random" was kept broad because serosurvey literature sources differed greatly in the extent to which the selection of individuals and villages was described. We introduced text to reflect this (line 165): "We only included survey studies that were designed to estimate the seroprevalence in the local community population."

We now include an Excel workbook that contains the dataset. The workbook contains a spreadsheet of "cleaned" data that are included in the model, as well as the original "raw" data from which the clean data were derived. The cleaned dataset contains a notes column that describes the survey to the extent that this information is provided in the original source.

* * *

Comment: What are the limitations of serosurveys in humans? For example, how good are the assays that measure previous infection and how long do antibodies last? Some information about the antibody response would be useful to inform interpretations of the yearly incidence estimates. If the authors' primary objective is to estimate the number of infections, I would assume that limitations in the measurement of past infection and assumptions about interpretations of these results should be discussed in detail – both in the methods and discussion section.

Response: We've incorporated more details of the LASV immune response in the manuscript:

- LASV antibodies are not necessarily specific to LASV. This is mentioned in the Data section at line 139: "Because arenavirus antibodies cross-react, a positive LASV antibody test in an individual rodent only indicates past infection with an arenavirus, and not necessarily LASV. In an effort to reduce the frequency of false positives in the training data, pixels that only contain LASV seropositive tests of rodents, and no positive LASV viral detection, were not used as training data."

- We assume that arenavirus antibodies do indicate past LASV infection in humans, as described at line 159: "As with the rodent LASV infection data, arenavirus antibodies are not specific to LASV. However, because human serosurveys were often conducted in LASV endemic areas or near documented locations of LASV-infected rodents, these serosurveys likely measured the fraction of humans with previous LASV infection, rather than exposure to another arenavirus."

- Details of immunity are discussed in the Methods section at line 302: "Studies suggest that LASV immunity is the result of a combination of antibodies and a cell-mediated immune response [7, 8]. Anecdotal cases have shown that LASV IgG antibodies can remain in the blood of individuals for decades [6]. However, other studies have indicated that the level of LASV antibodies, as well as the extent to which an individual is protected against subsequent LASV infection, can wane with time [7, 8]."

- Choices for antibody duration in the model are now more clearly specified at line 345: "In the first [scenario], any individual that has recovered from LASV infection remains seropositive for the remainder of their life ($\lambda = 0$ yr^{-1}). In the second scenario, seroreversion occurs at the rate estimated by McCormick et al. (1987) ($\lambda = 0.064$ yr^{-1}). In this case, an individual recovered from LASV is assumed to produce antibodies and maintain LASV immunity for an average duration of 15.6 years."

* * *

Comment: Since the resulting estimates of the numbers of cases each year from this analysis have a very wide range, might it be useful to conduct some sensitivity analyses around some of the key assumptions

about what a serologic response means? The authors correctly note that greater insight into the natural history of infection would be useful, but which points in particular would be most important to understand better for this kind of model?

Response: The greatest source of uncertainty in our model comes from not knowing the duration of time after infection that LASV antibodies and protective immunity are maintained. This is mentioned in the Discussion (line 460): "Using seroprevalence data from studies across West Africa, our model predicts between 892,800 – 4,359,800 LASV infections in humans occur each year. Where the true value lies within this range depends on whether or not seroreversion and subsequent LASV reinfection are regular features of human LASV epidemiology, and reinforces the need to better understand the scope for LASV reinfection."

3.4 Results

Comment: Figure 5: There is little variation in the predicted seroprevalence across West Africa – although the 'hotspots' have a predicted 18-20% seroprevalence, 12% prevalence in other areas also seems high. Most of the serosurveys used to inform this model have estimates that are outside of the entire predicted range.

Response: The modest fit of our regression is addressed at the top of this document.

* * *

Comment: Lines 340-341: It's unclear why the authors calculate estimated yearly infections using the as assumption of uniform risk.

Response: The uniform risk model has been removed. Addressed at the top of this document.

* * *

Comment: Lines 345-346: What assumptions were made about duration of antibody protection to estimate reinfections?

Response: These particular lines of text referred to the spatially uniform model which has been removed from the manuscript. The duration of antibody production in humans is addressed in a previous comment of Reviewer 3.

* * *

Comment: Additionally, information about the natural history of Lassa infections in humans would also be useful. How many infections are expected to be symptomatic? What proportion of people infected will die? Are there age or gender differences in risk or in seroprevalence? How were these accounted for in the model?

Response: We've added these details to the Results section (paragraph at line 387).

* * *

Comment: Predicted seroprevalence is 12% even in areas without the known reservoir host. This seems like a limitation in the ability of the model to predict human risk. Indeed, the fit of the model isn't great – would be good to include more about this in the discussion section.

Response: Addressed at the top of this document.

3.5 Discussion

Comment: This section could be improved by reducing the overlap with the results.

Response: We have kept this in mind while revising the manuscript.

* * *

Comment: Lines 383-385: Demographic and Health Surveys are routinely collected from West Africa and these include home building materials. These data are publicly available and could be incorporated into the model, as the authors suggest.

Response: We agree that the DHS datasets are a valuable resource for predictors that might modulate the amount of realized spillover into human populations. However, because of the extent of the DHS surveys, we feel that consideration of these additional factors deserves a separate manuscript. This manuscript sets up a framework that allows different sources of risk to be evaluated by their ability to explain spillover into humans, and quantifies the extent to which the distribution of LASV infected *M. natalensis* explains the spatial variation of arenavirus seroprevalence in humans. Future work will identify additional human-centric predictors that explain human spillover.

* * *

Comment: Lines 394-396: While the authors identified areas of relatively higher risk, or higher numbers of infections because of larger population sizes, it's unclear why other areas that still have significant risk shouldn't be targeted for surveillance and risk mitigation strategies. Why are relative differences in risk more important than absolute risk?

Response: We've changed this text so that this difference isn't highlighted. Instead, the paragraph (line 443) now describes which regions of West Africa should be prioritized for further surveillance.

* * *

Comment: I would be interested in hearing more from the authors about the modest correlations with the human seroprevalence data. Indeed, many of those studies are very old and it is likely that the conditions that drive Lassa transmission have changed in the intervening years. What are the pros and cons of the data used for the models and why is the correlation so modest? What else are we missing about this disease system?

Response: Addressed at the top of this document.

* * *

Comment: Lines 392-394: The authors mention multiple times the use of vaccines for rodents. As far as I know, there are no rodent vaccines for Lassa. There are efforts underway to develop human vaccines. It seems that part of the rationale for this study is to inform prevention; if true, it would be useful to provide a more informed discussion of the possible strategies and how their deployment might be facilitated by this research.

Response: We've added details describing vaccination campaigns in wildlife to the Discussion (paragraph at line 478).

* * *

Comment: Line 444: The authors bring up the idea of Lassa elimination which seems to be an unrealistic goal for a pathogen endemic in a wildlife host. Are there examples of elimination from other disease systems that the authors can reference to support this idea?

Response: We've added a reference (line 483) on the success of vaccination campaigns in red fox [14].

* * *

Comment: The authors state (Lines 467-469): "...the structured machine-learning models we develop here provide insight into what aspects of environment, reservoir, and virus, contribute to spillover, and the potential risk of subsequent emergence into the human population." It would be good to more completely discuss each of these points in terms of what was previously known and what we learned new from this study for each of these points to highlight the added value of this work.

Response: We've expanded the Discussion section to include text on each of these aspects. The paragraph at line 491 discusses the connection between the reservoir, virus, and the environment.

* * *

REFERENCES

1. Basinski AJ. Pathogen Spillover Forecast; 2020. Github repository https://github.com/54481andrew/pathogen-spillover-forecast.git.
2. Mylne AQ, Pigott DM, Longbottom J, Shearer F, Duda KA, Messina JP, et al. Mapping the zoonotic niche of Lassa fever in Africa. Trans R Soc Trop Med Hyg. 2015;109(8):483–492. doi:10.1093/trstmh/trv047.
3. WorldBank. Life expectancy at birth, total (years); 2020. https://data.worldbank.org/indicator/SP.DYN.LE00.IN.
4. Colangelo P, Verheyen E, Leirs H, Tatard C, Denys C, Dobigny G, et al. A mitochondrial phylogeographic scenario for the most widespread African rodent, *Mastomys natalensis*. Biol J Linn Soc. 2013;108(4):901–916. doi:10.1111/bij.12013.
5. Olayemi A, Oyeyiola A, Obadare A, Igbokwe J, Adesina AS, Onwe F, et al. Widespread arenavirus occurrence and seroprevalence in small mammals, Nigeria. Parasites Vectors. 2018;11(1):416. doi:10.1186/s13071-018-2991-5.
6. Bond N, Schieffelin JS, Moses LM, Bennett AJ, Bausch DG. A historical look at the first reported cases of Lassa fever: IgG antibodies 40 years after acute infection. Am J Trop Med Hyg. 2013;88(2):241–244. doi:10.4269/ajtmh.12-0466.
7. McCormick JB, Webb PA, Krebs JW, Johnson KM, Smith ES. A prospective study of the epidemiology and ecology of Lassa fever. J Infect Dis. 1987;155(3):437–444. doi:10.1093/infdis/155.3.437.
8. Fisher-Hoch S, Hutwagner L, Brown B, McCormick J. Effective vaccine for Lassa fever. Journal of virology. 2000;74(15):6777–6783. doi:10.1128/JVI.74.15.6777-6783.2000.
9. Fichet-Calvet E, Lecompte E, Koivogui L, Soropogui B, Doré A, Kourouma F, et al. Fluctuation of abundance and Lassa virus prevalence in *Mastomys natalensis* in Guinea, West Africa. Vector Borne Zoonotic Dis. 2007;7(2):119–128. doi:10.1089/vbz.2006.0520.

10. Gibb R, Moses LM, Redding DW, Jones KE. Understanding the cryptic nature of Lassa fever in West Africa. Pathog Glob Health. 2017;111(6):276–288. doi:10.1080/20477724.2017.1369643.

11. Peterson TA, Moses LM, Bausch DG. Mapping transmission risk of Lassa fever in West Africa: the importance of quality control, sampling bias, and error weighting. PLoS One. 2014;9(8):e100711. doi:10.1371/journal.pntd.0000388.

12. Hijmans RJ. Cross-validation of species distribution models: removing spatial sorting bias and calibration with a null model. Ecology. 2012;93(3):679–688. doi:10.1890/11-0826.1.

13. Jones KE, Patel NG, Levy MA, Storeygard A, Balk D, Gittleman JL, et al. Global trends in emerging infectious diseases. Nature. 2008;451(7181):990–993. doi:10.1038/nature06536.

14. Freuling CM, Hampson K, Selhorst T, Schröder R, Meslin FX, Mettenleiter TC, et al. The elimination of fox rabies from Europe: determinants of success and lessons for the future. Philos Trans R Soc Lond, B, Biol Sci. 2013;368(1623):20120142. doi:10.1098/rstb.2012.0142.
