# Supplementary material for: Bridging the gap: Using reservoir ecology and human serosurveys to estimate Lassa virus spillover in West Africa
Source: PLoS Comput Biol. 2021 Mar 3;17(3):e1008811. doi: 10.1371/journal.pcbi.1008811 (PMC7959400; doi:10.1371/journal.pcbi.1008811)
Supplement: Attachment — Submitted filename: ResponseToReviewers.pdf [file pcbi.1008811.s004.pdf]

Dear Dr. Amy Wesolowski and Dr. Nina Fefferman,

We are once again grateful for the helpful suggestions for improving our manuscript and for the opportunity to further revise our submission. All revisions to the datasets and code have been pushed to GitHub in the "rev2" branch of the pathogen-spillover-forecast repository [1]. Please note that we've slightly modified the title of our manuscript to "Bridging the gap: Using reservoir ecology and human serosurveys to estimate Lassa spillover in West Africa." In previous versions, the word "incidence" was used instead of "spillover." We feel that this change more accurately describes the estimates that our model produces.

We begin by addressing a key issue brought up in this most recent round of review. Next, we reply to the remaining comments that were emphasized by both the reviewer and editors. Then, we discuss changes made to the dataset. Finally, we address any remaining line-by-line comments of the reviewer.

A key concern raised in this round of review was the magnitude of our estimates for the annual number of human LASV infections. It is our belief that estimates of the total number of LASV infections in humans of West Africa may be much larger than conventional wisdom because 1) the health infrastructure necessary to diagnose Lassa Fever is lacking in much of West Africa, and 2) longitudinal seroconversion studies have suggested that the vast majority of all LASV infections are asymptomatic or mild, and 3) LASV reinfection may be a common phenomenon [2]. Because of this belief, we have incorporated Reviewer 1's helpful suggestion that we refer to our annual estimates as "LASV infections," rather than cases, which might imply LASV infection with symptoms. Our goal for this manuscript was to derive estimates of the number of human infections using data that describes the ultimate source of infection (i.e., LASV in *M. natalensis*), rather than being guided by reports of hospital records that are biased toward cases of Lassa Fever with severe symptoms. Although our estimates exceed some conventional annual LASV infection estimates, they are in line with another well-cited study that broadly estimates the number of LASV cases based on similar human serology data [3]. We've added this citation to the discussion (line 463) to help validate our estimates.

REVIEWER COMMENTS THAT WERE EMPHASIZED BY THE EDITOR

Comment: Address the use of SIRS dynamics over SIR

Response: Both the reviewer and editors critiqued our emphasis of the SIRS vs SIR models in the manuscript. We presented the SIRS model in the main text because 1) some studies suggest seroreversion occurs; and 2) the SIRS model is the more general model. Specifically, the model equations, as well as the equations derived throughout the Methods section, represent the SIR model when $\lambda = 0$. We feel it would be redundant to present these equations in the main text with $\lambda = 0$, and again in the supplemental with $\lambda \neq 0$. To help reduce emphasis on the SIRS dynamics, we changed text that referred to "SIRS model" in the following places:

- line 200: we changed "SIRS model" to "epidemiological model"
- line 277: we changed "SIRS model" to "an epidemiological model, based on the classic susceptible-infected-recovered framework,..."
- Figure 2: we changed "SIRS model" to "Epidemiological model"

* * *

Comment: Address the possibility of using age-seroprevalence data. Reviewer 1 states that "The authors note in their response that cell-mediated immunity is known to play a role in LASV response in humans

and that Abs wane with time—this may be true, but those dynamics are still not SIRS. If humans remain immune but seronegative, they should move into a different class that is Ab negative but certainly not susceptible; these dynamics could be modeled, and I'll emphasize again that information on the age structure of the serological response would be helpful in assessing this."

Response: We investigated the idea proposed by the reviewer and found that this addition does not change the LASV human infection estimates provided by our framework. Let C denote the class of individuals that maintain immunity to LASV after losing detectable levels of antibodies. A model that is consistent with the reviewer's suggestion is

$$\begin{aligned} \frac{dS}{dt} &= b - dS - FS + \lambda(1-\alpha)R, \\ \frac{dI}{dt} &= FS - dI - \gamma I, \\ \frac{dR}{dt} &= \gamma(1-\mu)I - dR - \lambda R \\ \frac{dC}{dt} &= \alpha\lambda R - dC. \end{aligned} \tag{1}$$

In the above model, a fraction α of individuals that lose detectable antibodies transition into class C, and a fraction $1-\alpha$ transition into the susceptible class.

In direct analogy with what is presented in the main text, steady-state analysis of System (1) can be used to estimate the annual number of LASV infections (i.e., the term FS^*) that are implied by a given seroprevalence. We've added these calculations in the Mathematica document and pdf output contained in the "Human LASV Incidence" subdirectory of the repository (section entitled "Model of Human LASV spillover with undetectable immune class"). The final expression for FS^* is unchanged from what we have in the main text (i.e., α does not influence the LASV case estimates). This occurs because our estimates are based on the number of annual cases (i.e., FS^*) that are implied by the predicted seroprevalence in a region. In our equations, it is true that increasing α decreases the number of humans that are susceptible (S) to LASV in a population. However, our estimation framework then predicts a higher force of infection F in order to compensate for this decrease in FS^*.

We agree that using age-seroprevalence data to assess different human epidemiological model structures is a relevant and fascinating project. At the same time, however, we feel that this analysis deserves its own manuscript. The current human model 1) provides the simplest connection from environmental risk to observed human seroprevalence, 2) is not limited to those serosurveys that report age-seroprevalence data, and 3) can be improved upon in future work.

MODIFICATIONS TO THE LASSA VIRUS DATASET

Reviewer 1 commented that some absences in our rodent LASV dataset might only be due to sampling having occurred in a season when LASV prevalence is low, and requested that sampling-date information be available in the dataset. This is a fair point, and we have taken steps to provide information on when rodent sampling took place for each entry in the clean LASV dataset, in those cases where this information is provided by the primary literature source. Specifically, we've added a column, "Survey Dates", that describes the years and months of rodent sampling. When year-month is not available in the literature source, we included the season (wet or dry) and year of sampling.

In addition to this change, we made other small corrections to the dataset that resulted in 4 fewer absences. First, we added seroprevalence data from Coulibaly, N'Golo (2009), which had previously been overlooked. Previously, only the PCR testing data in Coulibaly, N'Golo (2009) were included in the dataset. The

addition of immunity data changed 3 sites that were previously classified as absences into ambiguous sites. Finally, we reorganized entries of trapping data from the manuscripts Lecompte, Fichet-Calvet (2006) and Fichet-Calvet, Becker-Ziaja (2014). These studies report PCR and serology tests, respectively, on the same group of rodents. As such, the data from these papers is best organized into a single row for each site. This change brought to light a sampled site, Kodoko, that was being double counted in our dataset. These changes did not have a substantial impact on the resulting models or LASV infection estimates.

REVIEWER 1

1.1 Major Comments

Comment: Lack of seasonality included in either the reservoir or pathogen layer: point taken that dynamics are assessed over long timescales and that spatial, environmental factors are accounted for. However, given the sparseness of the data for some of these localities, as well as the short lifespan of the rodent hosts (meaning Ab+ data may be difficult to acquire), I still think it is possible that a few rodents are sampled at the wrong time of year to get a false negative LASV pixel result for a given region. Along these lines, do you really think 5 Ab negative rodents is enough to conclude that a site is LASV(-)? My understanding is that the average lifespan of M. natalensis is only 6 months, so I would not be surprised at all to find 5 seronegative individuals in a site where Lassa really does occur. Can you provide a baseline seroprevalence in rodents of a LASV(+) site for comparison?

Response: Across the sites that are 1) deemed LASV+ by our definition, and 2) have seroprevalence data in rodents, the average seroprevalence is 24%. That said, we agree that there might be some false-negatives present in our dataset. However, because we bootstrap our model fitting procedure, we encourage the LASV sub-model to learn robust patterns that are not dependent on absences or presences within any particular region. Consequently, the LASV layer can still learn general trends in overall LASV prevalence so long as false negatives are not too prevalent. Ultimately, finding out the true distribution of LASV in rodents will require more longitudinal sampling effort.

Because of the reviewer's comments, we reran the full model with the minimum number of rodents required in the definition of a LASV-absence set to ten. This results in two fewer absences: one from Guinea, and one from Nigeria. All of the absences within central West Africa remain. Consequently, the general prediction of the model remains similar, with precipitation contingency being the primary driver of LASV distribution and the distribution of LASV concentrated in western and eastern West Africa. The additional simulations have been uploaded to GitHub.

* * *

Comment: I like the Excel workbooks with the raw data added to the Github repository, but neither the M. natalensis nor the Lassa tables report month or season of each data point. If these data exist, they should be reported and, ideally, included in the regression model for the reservoir and pathogen layers. At a very minimum, I would like the authors summarize the seasonality of input data in some way to show that there is not some glaring inconsistency whereby a rodent was never sampled at the time of year relevant for the disease in question in a particular area.

Response: We've incorporated the reviewer's suggested changes to the dataset. The table below summarizes the rodent surveys that define our absence sites. At least for many of the absences in central West Africa (e.g., Ghana, Ivory Coast), surveying was conducted in the wet season (roughly April - October) when LASV prevalence is believed to peak [4]. Other absences do not have as thorough of

sampling. However, we keep these in the dataset because they accurately portray what is currently known about LASV prevalence in rodents for those regions, and because our bootstrapping procedure encourages our model to learn robust relationships that do not depend heavily on specific rodent surveys.

**Country**	**Survey Dates**	**# Tested Ab**	**#Tested Virus**	**Reference**
Ghana	2010: wet; 2011: wet	0	33	[5]
Ghana	2010: wet; 2011: wet	0	59	[5]
Ghana	2010: wet; 2011: wet	0	14	[5]
Ghana	2010: wet; 2011: wet	0	10	[5]
Ghana	2010: wet; 2011: wet	0	32	[5]
Ghana	2010: wet; 2011: wet	0	40	[5]
Ghana	2010: wet; 2011: wet	0	17	[5]
Guinea	2004: February	24	24	[6]
Guinea	2004: February	70	70	[6]
Guinea	2003: May, October	11	11	[6, 7]
Guinea	2004: February	9	9	[6]
Guinea	2004: January	14	14	[6]
Guinea	2005: February	0	35	[6]
Ivory Coast	2003: December; 2004: March, November, December; 2005: March, August, October, November	97	97	[8]
Ivory Coast	2003: December; 2004: March, November, December; 2005: March, August, October, November	164	164	[8]
Ivory Coast	2003: December; 2004: March, November, December; 2005: March, August, October, November	12	12	[8]
Mali	2004: February	39	0	[7]
Mali	2009: June	0	17	[9]
Mali	2009: June	0	40	[9]
Mali	2011: October	22	22	[10]
Mali	2011: October	11	11	[10]
Mali	2012: March	11	11	[10]
Mali	2012: March	33	34	[10]
Nigeria	2012: March	7	7	[11]
Nigeria	2011: January	13	13	[11]
Nigeria	2011: dry, wet; 2012: dry, wet	34	34	[11]
Sierra Leone	NA	19	8	[2]

Table 1: The column "Survey Dates" indicates when rodent sampling took place. The format is "Year1: month1, month2, etc; Year2: month1, month2, etc. " In some primary sources, only season (wet or dry) was provided. NA indicates that no time-of-sampling information was provided by the original publication.

* * *

Comment: Concern over the emphasis on SIRS dynamics over SIR with very little support for this decision. The authors attempt to address these concerns, and in fact, they do a decent job of emphasizing that support is fairly weak for SIRS assumptions in the results and discussion; however, the methods are still entirely focused on an SIRS approach and now inconsistent with the rest of the paper. This discrepancy needs to be addressed – see specific line by line comments below.

Response: Addressed at the top of this document and in line by line comments below.

* * *

Comment: Additionally, the authors note in their response that cell-mediated immunity is known to play a role in LASV response in humans and that Abs wane with time—this may be true, but those dynamics are still not SIRS. If humans remain immune but seronegative, they should move into a different class that is Ab negative but certainly not susceptible; these dynamics could be modeled, and I'll emphasize again that information on the age structure of the serological response would be helpful in assessing this.

Response: Addressed at the top of this document.

* * *

Comment: One other point that I noted on my re-read was that the authors spend a lot of time at the end of the paper predicting annual "cases" (e.g. Fig 6). Perhaps I've become too steeped in this difference from COVID, but I would advocate for changing the terminology to "infections" rather than "cases" which to me, implies symptoms.

Response: This is an excellent point and we have incorporated this suggestion.

1.2 Abstract

Comment: No need to address this in the abstract necessarily, but you mention 'West Africa' throughout the manuscript and show a map of the UN-defined region in all of your figures. It would be helpful to formally define this region (or cite a UN source) somewhere in the text so that the geographic extent of analysis does not seem arbitrary.

Response: We've added a citation of UN geographical regions (line 73) [12].

* * *

Comment: Additionally, I think the 4 million (+) annual infections from the SIRS model is a fairly unreasonable projection, and I would suggest to leave this finding out of the abstract.

Response: We've removed reference of this number from the abstract.

1.3 Author Summary

Comment: Is Nigeria truly at risk for emergence of "new strains" of Lassa virus or just at risk for 'emergence'? The authors do not report any evidence as to what genotypes to expect in one reason vs. another.

Response: We've removed the reference to "new strains."

1.4 Main paper

Comment: Lines 148-153: given the short lifespan of M. natalensis and the seasonal dynamics of Lassa, it seems that 5 seronegative rodents might be easy to acquire. What is the comparative seroprevalence in Lassa-positive regions?

Response: Addressed above.

* * *

Comment: Line 163-167: It would be would be helpful to see a PRISMA diagram in the supplement that explains how you compiled your data for each layer: what terms were searched and surveys were excluded at each point in the analysis. You are very clear about the search terms used for the rodent infections—and you include the helpful Workbooks on Github—but less so here for the humans, and I can't find the raw human data in the repository. What terms were searched and what serosurveys were excluded at each point in the analysis?

Response: Our Data section and Excel spreadsheets describe where our data sources come from and what data were included. The human dataset originates from the same searches as the rodent dataset. The search keywords were kept broad so as not to limit to one species or the other. This is specified at line 120 and line 156.

The raw human data are in the same Excel document as all of the other Lassa data. Specifically, raw human data are those entries in the "Raw Lassa Literature" spreadsheet with Genus column equal to "Homo" and species column equal to "sapiens." We've added text to the README file on GitHub to clarify this.

* * *

Comment: Table 3: Edit % Pos. to % Seropositive. Edit table title to "serosurvey" instead of "survey"
Response: We've incorporated this suggestion.

* * *

Comment: Line 201/Figure 2/Line 278: Why do we assume SIRS as default? In the Results, you report under SIR assumptions but not mention is made of this in the Methods.
Response: We do not mean to emphasize the SIRS dynamics nor claim that $\lambda > 0$ with certainty. We present the SIRS model in the main text because the SIR model is a submodel of the SIRS model with $\lambda = 0$. Explaining both models and count-derivations separately would be redundant. As stated at the top of this document, we've modified the wording to reduce the emphasis on SIRS dynamics at the locations pointed out in the reviewer's comment.

* * *

Comment: Line 307: Again, what about the alternative version in which immunity is maintained?
Response: An alternative version of our model is considered at the top of this document. We do not include this alternative version because it does not modify our estimates of human Lassa infection.
In addition, we've moved the text that describes assumptions of LASV immunity into its own paragraph. In that paragraph (line 303), we mention that the duration of LASV immunity is not fully understood. We also explicitly mention that immunity is lifelong in the case that $\lambda = 0$.

* * *

Comment: Also, as mentioned above transition to a cell-mediated immune class should not give an SIRS-like dynamic, as individuals who wane from the R class will not move back to S
Response: Addressed at the top of this document.

* * *

Comment: Line 340: Again, you emphasize this rate of seroreversion extensively. I would suggest presenting the uncertainty in this rate—and the two different models derived from that uncertainty at the beginning of this methods section to make it clear that two possible outcomes (and a range in between them) are present.
Response: We feel that starting this subsection with an overview of what is known about LASV immunity in humans would feel out of order. The presentation of the epidemiological model is much more natural when the reader is walked through the state variables and processes that are associated with susceptible, infected, then recovered individuals. The point about seroreversion then naturally comes up when discussing the assumptions surrounding the recovered class. The uncertainty in the seroreversion rate parameter naturally comes up after the model equations, when the values for the model parameters are described (line 342). In that paragraph, we clearly present the two scenarios, lifelong immunity and waning immunity, that are being investigated.

* * *

Comment: Line 372: As mentioned above, I would like to know if season of sampling for rodents influences the Lassa risk map.

Response: Addressed above.

* * *

Comment: Why do you think Lassa is restricted in the west and east if not as a result of human density? Can you discuss this in the Discussion?

Response: This is an interesting but still unresolved question. Our machine learning framework suggests that the occurrence of LASV in rodents is most consistent with rainfall patterns like precipitation contingency. The central portion of West Africa has lower precipitation contingency than the at-risk regions of Nigeria, Sierra Leone, and Guinea, therefore the probability of LASV presence is also lower. We've added text at line 508 in the Discussion that mentions the possibility that LASV survives better outside of a host in wetter environments.

* * *

Comment: Fig 4. What is shown on the x-axis? Probability of a given pixel being LASV (+)? If so, label as such.

Response: The x-axis of Figure 4 shows the combined risk layer, notated D_X in the manuscript. We changed the label to "Predicted Risk of Lassa (D_X)" to avoid confusion with the D_L risk layer that describes the probability that Lassa virus is in *M. natalensis*, given that the rodent is present.

* * *

Comment: Fig 5. You only show serosurveys with pop sizes greater than 50 – why is this?

Response: The binomial regression weights each seroprevalence datapoint by the number of individuals tested in the serosurvey (line 275). We originally omitted the smaller serosurveys from the map in an attempt to make the residual datapoints easier to see and also because they contribute less to the coefficients estimated by the regression. We've revised the figure to include all serosurvey datapoints.

* * *

Comment: According to Table 3, it looks like some of these surveys must have had very few individuals tested – how did you account for this in your model? This is not clear from the supplement or the main text.

Response: The reviewer is correct that sites differ in the number of individuals tested. In the quasi-binomial regression, each seroprevalence estimate is weighted by the number of individuals that were tested. This method of "weighting" data is standard practice in binomial-structured models of prevalence. As a result, serosurvey locations that tested more individuals have a greater effect on the coefficients estimated by the regression. This is mentioned at line 275 in the main text. To emphasize this point, we also added mention of it at lines 89 and 98 of the Appendix.

In the previous draft, the column "# Tested" in Table 3 showed the *average* number of individuals tested per site sampled in that row's publication. This was mentioned in the table caption, however we realize now it is confusing given the column header. We've changed this column to instead present the total number of individuals tested across all sites in the focal publication.

* * *

Comment: Line 388: As mentioned above, I would suggest trading "cases" (implying symptomatic cases) with "infections" that might go unnoticed and explain some of these results. This terminology persists throughout the following paragraph

Response: We've incorporated this suggestion.

* * *

Comment: Table 4: Suddenly, all assumptions here switch to SIR when previously the paper emphasized SIRS. This distinction needs to be clarified and consistent throughout. I would suggest presenting results for SIR assumptions only and then including SIRS results in the supplement

Response: Addressed at the top of this document.

REFERENCES

1. Basinski AJ. Pathogen Spillover Forecast; 2020. Github repository https://github.com/54481andrew/pathogen-spillover-forecast.git.

2. McCormick JB, Webb PA, Krebs JW, Johnson KM, Smith ES. A prospective study of the epidemiology and ecology of Lassa fever. J Infect Dis. 1987;155(3):437–444. doi:10.1093/infdis/155.3.437.

3. Richmond JK, Baglole DJ. Lassa fever: epidemiology, clinical features, and social consequences. BMJ. 2003;327(7426):1271–1275. doi:10.1136/bmj.327.7426.1271.

4. Fichet-Calvet E, Lecompte E, Koivogui L, Soropogui B, Doré A, Kourouma F, et al. Fluctuation of abundance and Lassa virus prevalence in *Mastomys natalensis* in Guinea, West Africa. Vector Borne Zoonotic Dis. 2007;7(2):119–128. doi:10.1089/vbz.2006.0520.

5. Kronmann KC, Nimo-Paintsil S, Guirguis F, Kronmann LC, Bonney K, Obiri-Danso K, et al. Two novel arenaviruses detected in pygmy mice, Ghana. Emerg Infect Dis. 2013;19(11):1832. doi:10.3201/eid1911.121491.

6. Lecompte E, Fichet-Calvet E, Daffis S, Koulémou K, Sylla O, Kourouma F, et al. Mastomys natalensis and lassa fever, West Africa. Emerg Infect Dis. 2006;12(12):1971. doi:10.3201/eid1212.060812.

7. Fichet-Calvet E, Becker-Ziaja B, Koivogui L, Günther S. Lassa serology in natural populations of rodents and horizontal transmission. Vector Borne Zoonotic Dis. 2014;14(9):665–674. doi:10.1089/vbz.2013.1484.

8. Coulibaly-N'Golo D, Allali B, Kouassi SK, Fichet-Calvet E, Becker-Ziaja B, Rieger T, et al. Novel arenavirus sequences in *Hylomyscus* sp. and *Mus (Nannomys) setulosus* from Côte d'Ivoire: implications for evolution of arenaviruses in Africa. PloS One. 2011;6(6):e20893. doi:10.1371/journal.pone.0020893.

9. Safronetz D, Lopez JE, Sogoba N, Traore SF, Raffel SJ, Fischer ER, et al. Detection of Lassa virus, Mali. Emerg Infect Dis. 2010;16(7):1123. doi:10.3201/eid1607.100146.

10. Safronetz D, Sogoba N, Lopez JE, Maiga O, Dahlstrom E, Zivcec M, et al. Geographic distribution and genetic characterization of Lassa virus in sub-Saharan Mali. PLoS Negl Trop Dis. 2013;7(12). doi:10.1371/journal.pntd.0002582.

11. Olayemi A, Obadare A, Oyeyiola A, Fasogbon S, Igbokwe J, Igbahenah F, et al. Small mammal diversity and dynamics within Nigeria, with emphasis on reservoirs of the Lassa virus. System Biodivers. 2018;16(2):118–127. doi:10.1080/14772000.2017.1358220.

12. United Nations. Geographical Regions; 2020. https://unstats.un.org/unsd/methodology/m49/#geo-regions.
